# Ophthalmic Notes—Cases from Practice

**Published:** 1874-09-01

**Authors:** A. D. Williams

**Affiliations:** St. Louis, Mo.


### MEDICAL EXAMINER.

A

*Semi-Monthly Journal of Medical Sciences.*

EDITED BY N. S. DAVIS, M.D., AND F. H. DAVIS, M.D.

No. XVII. CHICAGO, SEPT. 1, 1874. VOL. XV.

### Original Communications.

### OPHTHALMIC NOTES—CASES FROM PRACTICE.

By A. D. WILLIAMS, M.D., ST. LOUIS, MO.

A GENTLEMAN was treated four or five weeks by a prominent surgeon in Louisville for supra-orbital neuralgia, but without success. At the end of that time, the man had occasion to go to St. Louis, and continuing to suffer, he called in his family physician, who also treated him several days for supra-orbital neuralgia, but likewise without success. In the meantime his eye became red, and his physician was kind enough to refer him to me.

Upon examination I found the smallest crystal of white sand bedded in the surface of the cornea. Its removal put an end to all his supra-orbital neuralgia.

When the patient saw the foreign body, he expressed a doubt that such a small thing could cause him so much and long suffering. But the result proved it to be even so.

Case 2.—Mr. D.—For two weeks during this hot weather, particularly during the hottest part of the day, his eyes have been a source of great annoyance to him. They were not particularly painful, but he would frequently have to stop on the street and close them for a short time, apparently to rest them. Then he could move on a distance and again stop and close them, and so on.

I found a very small particle of coal sticking in the surface of one cornea. After its removal the trouble disappeared at once.

Case 3.—Mr. P., a railroad laborer, was cutting railroad iron with a cold chisel. Some small substance struck him in the right eye hard enough to knock him down. As soon as he got up he noticed that his eye was almost totally blind. It soon began to pain him severely, and continued to do so for three weeks. During these three weeks many physicians in the neighborhood of Springfield, Illinois, prescribed for him. In the meantime he fell into the hands of "The Twin Brothers," in Decatur. They, quack-like, promised a sure cure for so much money. The money was forthcoming, but the *cure* failed to come forth. At the end of three weeks the eye had ceased to pain him, but it remained practically blind—could only see fingers in some directions. So the man concluded to try his luck in St. Louis. His money being out (the Twins captured the most of it), he went to the City Dispensary and consulted the physician, who represents the board of health. He barely looked at him, and prescribed a solution of iodide of potassium: to take a teaspoonful three times a day, and return in three weeks. This did not satisfy the man, so he sought further advice.

Upon examination, I found that the eye was as bright as the other, not in the least red. The iris was discolored from iritis, but that had subsided. The patient could see fingers in some directions. The eye, as I have already stated, was not painful, and had not been so for several days.

The history of the case made me suspect that a piece of iron had gone into the eye. Near the equator of the ball, on its outer surface, I discovered a small black point, under the conjunctiva, about as large as a pin's head. Feeling this with the end of a probe, I found that it was a foreign body. I supposed it was a small piece of iron lying under the conjunctiva. I cut through the overlying conjunctiva, and got hold of the thing, but was not able to pull it out with the forceps. The effort to draw it out was intensely painful to the patient. I gave him chloroform, and made another effort to remove it with a larger pair of forceps, but without success. I found that the piece of iron had penetrated the sclerotic, and that had grown tightly around it, so as to hold it firmly. As I pulled the next time, I passed a knife in along the foreign body, and incised the sclerotic so as to enlarge the opening and allow it to slip out. This time I succeeded in removing it, and I must say that when it came out I was no little surprised at its unusual size. It proved to be over three-fourths of an inch long and one-fourth thick. It is remarkable that such a large, rough, ugly piece of iron could remain in the eye without causing very serious trouble. This had been in the eye for thirty days. For three weeks it was quite painful, but for several days previous to the time I saw him there had been no pain, and there was not even redness. The patient did not know, nor even suspect, that anything was in his eye. He supposed that some large object had struck him over the eye and fallen away. I should have observed above that the ophthalmoscope revealed nothing, as the whole interior of the eye was black from the escape of blood into the vitreous chamber. The only evidence of any thing being in the eye was the little black point under the conjunctiva, mentioned above. The piece of iron had entered endwise between the lids, without injuring either, and had passed upwards, backwards, and inwards, the internal end probably penetrating the inner wall of the eye.

There was hardly any redness following the removal of the foreign body. In seven or eight days the man left the city and went to work.

The vision was practically lost on account of the clouding of the vitreous humor by the escape of blood into it. The man could see fingers when he left. The form and appearance of the eye would be preserved.

In connection with this case I may barely mention an interesting case I had some years ago at Cincinnati:

The premature discharge of a blast in Ireland sent a fragment of blue lime-stone into a man's eye, and blinded it instantly. He suffered uninterrupted pain for sixteen years, during which time Sir Wm. Wylde operated on the other eye for cataract, but discovered nothing in the injured eye.

He came to me to know if any thing could be done for his operated eye, as he could not see well enough to work.

I found a piece of blue lime-stone in the injured eye, about the same length but thicker than this piece of iron. The stone had gone in sideways between the lids, and buried itself in the interior of the eye. The eyeball had gradually atrophied to a small stump, from the front of which one side of the stone was projecting between the lids. I loosened it by incising the sclerotic around it, and had no trouble to remove it. The man was no little surprised to see what a stone came out of his eye. The prospect of getting rid of the severe pain he had suffered for sixteen long years caused him to give such expressions of joy as can come only from an Irishman.

It is very strange that Wylde did not discover the stone at the time he operated on the other eye for cataract.

